# Incremental Diagnostic Value of Cell-Block-Based Targeted BRAF and RAS Testing in TBSRTC Category III–IV Thyroid Nodules: A Single-Center Retrospective Study

**DOI:** 10.3390/diagnostics16193258

**Published:** 2026-10-08

**Authors:** Cumhur Selçuk Topal, Yaşar Kemal Duymaz, Büşra Sönmez, Hanife Şeyda Genç

**Affiliations:** 1 Department of Pathology, Sultan II. Abdülhamid Han Training and Research Hospital, Tıbbiye Avenue, Üsküdar, 34668 Istanbul, Türkiye; 2 Department of Otorhinolaryngology, Sultan II. Abdülhamid Han Training and Research Hospital, Tıbbiye Avenue, Üsküdar, 34668 Istanbul, Türkiye; dyasarkemal@gmail.com (Y.K.D.); busrasonmez285@gmail.com (B.S.); 3 Department of General Surgery, Sultan II. Abdülhamid Han Training and Research Hospital, Tıbbiye Avenue, Üsküdar, 34668 Istanbul, Türkiye; hanifeseydagenc@gmail.com

**Keywords:** thyroid fine-needle aspiration, TBSRTC, BRAF, RAS, Sanger sequencing, cell block, NIFTP

## Abstract

**Background/Objectives:** Risk stratification of thyroid nodules classified as categories III–IV in The Bethesda System for Reporting Thyroid Cytopathology (TBSRTC) remains challenging. This study evaluated the incremental diagnostic value of cell-block-based targeted molecular testing beyond TBSRTC cytology within the 2023 TBSRTC and WHO 2022 frameworks. **Methods:** This retrospective study included 126 TBSRTC category III–VI thyroid fine-needle aspiration samples. BRAF exon 15 and KRAS/NRAS exons 2–4 were assessed by bidirectional Sanger sequencing; TERT testing was limited to six selected cases. Histopathology was available for 69 nodules, including 59 TBSRTC category III–IV nodules in the primary diagnostic-performance cohort. Logistic regression, ROC analysis, bootstrap correction, repeated stratified five-fold cross-validation, calibration, and exploratory decision curve analysis were performed. **Results:** BRAF/KRAS/NRAS mutations were detected in 35 of 126 nodules (27.8%). Among resected nodules, mutations occurred in 23 of 32 malignant (71.9%) and 10 of 37 benign/NIFTP cases (27.0%; *p* < 0.001); BRAF showed the strongest association with malignancy (50.0% vs. 2.7%; *p* < 0.001). In the resected cohort, combined cytology and molecular testing yielded an AUC of 0.81 versus 0.70 for cytology alone (bootstrap ΔAUC 0.10; 95% CI, 0.03–0.18). In the surgically verified, surgery-enriched TBSRTC category III–IV subgroup (*n* = 59), the combined model improved AUC from 0.65 to 0.81 (bootstrap ΔAUC 0.15; 95% CI, 0.01–0.29) and achieved 57.7% sensitivity, 90.9% specificity, 83.3% positive predictive value, and 76.3% accuracy. **Conclusions:** In the surgically verified TBSRTC category III–IV subgroup, targeted BRAF/RAS testing provided incremental rule-in information beyond cytology but had limited rule-out capability. BRAF V600E was strongly associated with malignancy, whereas non-V600 BRAF and RAS alterations required morphologic and clinical context. Negative molecular results should not be used in isolation to exclude malignancy. External validation is required.

## 1. Introduction

Fine-needle aspiration (FNA) cytology plays a central role in the evaluation of thyroid nodules; however, accurate preoperative risk stratification remains challenging for nodules with indeterminate cytology [1].

Despite its high diagnostic value, approximately 20–30% of thyroid FNA samples are classified as indeterminate, particularly those classified as category III (atypia of undetermined significance) or category IV (follicular neoplasm/suspicious for follicular neoplasm) in The Bethesda System for Reporting Thyroid Cytopathology (TBSRTC) [2], creating a persistent diagnostic challenge. These categories show overlapping risks of malignancy and frequently lead to diagnostic surgery, although many resected nodules prove benign [3].

The 2023 update of TBSRTC and the WHO 2022 classification have refined malignancy-risk estimates and incorporated contemporary histomolecular concepts [2,4]. These developments have further highlighted the limitations of cytomorphology alone and the need for adjunctive diagnostic tools.

Molecular testing has therefore emerged as an important adjunct to cytology. *BRAF* V600E is strongly associated with papillary thyroid carcinoma and functions primarily as a highly specific rule-in marker [5].

KRAS and NRAS mutations occur across a spectrum of follicular-patterned lesions, including adenomas, noninvasive follicular thyroid neoplasm with papillary-like nuclear features (NIFTP), and carcinomas, and consequently require interpretation in morphologic and clinical context [6].

Recent studies have shown that targeted and next-generation sequencing-based molecular testing can improve diagnostic performance in indeterminate thyroid nodules [3,7,8]. At the same time, recognition of NIFTP as an indolent, low-risk neoplasm has altered malignancy-risk estimates and emphasized the importance of integrating molecular findings with definitive histomorphology [9].

Molecular testing may also support risk-adapted surgical decision-making when interpreted within guideline-defined clinical and cytomorphological contexts [10,11,12]. A recent large Turkish tertiary-center study demonstrated the feasibility and high specificity of low-complexity BRAF/RAS testing using fresh FNA washout specimens [13]. Unlike that specimen-feasibility study, the present analysis evaluates cell-block-based testing and quantifies the incremental value beyond TBSRTC cytology through multivariable discrimination, internal validation, calibration, and decision curve analysis within the updated WHO 2022 and 2023 TBSRTC frameworks.

Accordingly, the primary aim of this study was to determine whether targeted BRAF, KRAS, and NRAS mutation testing provides incremental diagnostic value beyond TBSRTC cytology for predicting final histopathology in surgically verified TBSRTC category III–IV thyroid nodules. Secondary aims were to characterize mutation distribution across TBSRTC categories and to evaluate the discrimination, calibration, internal validity, and exploratory clinical utility of the combined cytology–molecular model. We hypothesized that the addition of molecular results would improve rule-in performance, particularly specificity and positive predictive value, compared with cytology alone.

## 2. Materials and Methods

### 2.1. Study Design and Patient Selection

This single-center retrospective study identified thyroid FNA cases from the institutional pathology archive between January 2016 and December 2024 by applying the following eligibility criteria: adequate cytological material, classification as TBSRTC category III–VI, and available BRAF/KRAS/NRAS molecular results. The resulting cohort comprised 126 eligible nodules. Clinical and demographic data, including age, sex, and nodule diameter, were obtained from electronic medical records. Surgical histopathology was available for 69 nodules; the remaining 57 nodules had no resection specimen and were excluded from diagnostic-performance analyses. The primary diagnostic-performance cohort comprised 59 surgically verified TBSRTC category III–IV nodules, whereas the remaining 10 resected TBSRTC category V–VI nodules were included only in secondary analyses of the entire resected cohort. No case showed clinical or cytomorphological features suggestive of medullary thyroid carcinoma. The targeted panel was designed primarily for follicular cell-derived thyroid nodules and was not intended as a comprehensive classifier for all thyroid malignancies.

Molecular testing results were available to the treating clinicians before decisions regarding surgical management. Resection was not determined by a predefined study protocol but was decided during routine clinical care by integrating the TBSRTC cytologic category, molecular findings, ultrasonographic features, and other relevant clinical findings. Consequently, histopathologic verification was available for a clinically selected subset of nodules, and the likelihood of resection was not independent of the index molecular test.

### 2.2. Cytological Evaluation

FNA samples were prepared using conventional smears and/or liquid-based cytology. All cases were reviewed and classified according to the 2023 edition of The Bethesda System for Reporting Thyroid Cytopathology (TBSRTC) [2]. The study cohort comprised the following categories:

- Category III: Atypia of undetermined significance (AUS)
- Category IV: Follicular neoplasm/suspicious for follicular neoplasm (FN/SFN)
- Category V: Suspicious for malignancy
- Category VI: Malignant

Cytological evaluation was performed by experienced cytopathologists.

### 2.3. Molecular Analysis

Molecular analysis was performed on formalin-fixed, paraffin-embedded cell-block material prepared from thyroid fine-needle aspiration samples. For each case, the corresponding hematoxylin and eosin-stained section was reviewed, and the area containing the highest proportion of lesional follicular cells was selected. Samples were considered suitable for molecular analysis when the estimated lesional cell fraction was at least 25%. Selected lesional areas were manually macrodissected with a scalpel from ten unstained sections, each 10 µm thick.

Following standard deparaffinization and rehydration, genomic DNA was isolated using the QIAamp DNA FFPE Tissue Kit (Qiagen, Hilden, Germany). DNA concentration was measured spectrophotometrically using a NanoDrop instrument. Exons 2, 3, and 4 of KRAS; exons 2, 3, and 4 of NRAS; and exon 15 of BRAF were amplified by polymerase chain reaction using HotStarTaq DNA Polymerase (Qiagen) and gene-specific primers in a thermal cycler.

Each sample was tested in duplicate together with positive, negative, and no-template controls. Amplification efficiency and potential contamination were evaluated by gel electrophoresis. PCR products were purified using the QIAquick PCR Purification Kit (Qiagen). Bidirectional Sanger sequencing was performed using forward and reverse primers and the BigDye Terminator v3.1 Cycle Sequencing Kit (Applied Biosystems, Foster City, CA, USA). Following ethanol precipitation, sequencing products were analyzed on an ABI 3730 48-capillary DNA Analyzer.

Electropherograms were evaluated both visually and using SeqScape Software version 3.0 by comparison with the reference sequences NM_004985.4 for KRAS, NM_002524.4 for NRAS, and NM_004333.4 for BRAF. Sequence variants were accepted only when supported by adequate bidirectional sequencing quality and appropriate control results. All 126 included cases had been tested for BRAF, KRAS, and NRAS; no molecular result was missing. Database cells without a recorded variant were verified as negative and coded accordingly. Because of the analytical characteristics of Sanger sequencing, mutations may not be detected in samples containing less than 25% lesional cells; therefore, a negative result does not completely exclude a low-level mutation. TERT promoter variants C228T and C250T were assessed in a clinically selected subset of six cases. Because TERT testing was not performed systematically, the primary molecular predictor used in the diagnostic models was positivity for any BRAF, KRAS, or NRAS mutation; TERT findings were analyzed descriptively.

### 2.4. Histopathological Evaluation

Surgical specimens were evaluated according to the 2022 WHO classification of thyroid tumors [4]. Final histopathology was dichotomized as malignant versus benign/NIFTP for diagnostic-performance analysis. For these analyses, NIFTP was considered non-malignant and included in the benign/NIFTP outcome group, consistent with its low-risk biological classification in the WHO 2022 framework. NIFTP was diagnosed using established criteria, including an encapsulated or well-demarcated follicular growth pattern, papillary thyroid carcinoma-type nuclear features, absence of capsular and vascular invasion, and absence of true papillae [9].

### 2.5. Statistical Analysis

Continuous variables are reported as median (interquartile range [IQR]) and were compared using the Mann–Whitney U test. Categorical variables are reported as n (%) and were compared using Pearson’s chi-square test when expected cell frequencies were ≥5, Fisher’s exact test for 2 × 2 tables with small expected frequencies, or the Fisher–Freeman–Halton exact test for larger contingency tables with small expected frequencies.

The diagnostic performance of cytology, molecular testing, and their combination for predicting final histopathology (malignant vs. benign/NIFTP) was evaluated using binary logistic regression. Because TBSRTC is an ordinal categorical classifier rather than a linear scale, the TBSRTC category was modeled primarily as a categorical variable, dummy-coded with category III as the reference. Analyses using binary coding (TBSRTC category III vs. IV–VI) and continuous coding were performed as sensitivity analyses. For the primary TBSRTC category III–IV combined model, regression coefficients, odds ratios (ORs), 95% confidence intervals (CIs), and Wald *p*-values were reported.

Model-derived predicted probabilities were used for ROC analysis. Discrimination was quantified using the area under the ROC curve (AUC) with 95% confidence intervals. Incremental discrimination of the combined model versus cytology alone was quantified by the paired AUC difference with a stratified nonparametric bootstrap confidence interval (5000 resamples). Internal validation comprised 500 stratified bootstrap resamples for optimism correction and repeated stratified five-fold cross-validation (100 repeats); five folds were chosen to preserve adequate numbers of malignant and non-malignant cases within each validation fold. Calibration was summarized using calibration plots, the Brier score, and a Hosmer–Lemeshow-type test with five risk groups. Sensitivity, specificity, positive predictive value (PPV), negative predictive value (NPV), and accuracy were calculated at the Youden-optimal cutoff derived in the same dataset and are therefore descriptive. Clinical utility was explored using decision curve analysis; interpretation focused on threshold probabilities from 0.20 to 0.50 and was considered hypothesis-generating. A two-sided *p*-value < 0.05 was considered statistically significant. Analyses were performed in jamovi 2.7 using the DiagROC R package and in Python 3 using scikit-learn and SciPy. Random resampling used a fixed seed (20260325). Reporting and interpretation followed STARD and TRIPOD principles; the internal-validation, calibration, and decision-curve procedures were informed by established methodological guidance [14,15,16,17,18].

### 2.6. Ethical Approval and Informed Consent

The study was conducted in accordance with the Declaration of Helsinki and was approved by the Ethics Committee of Sultan II. Abdülhamid Han Training and Research Hospital (protocol code E-54230385-929-301089224; Ethics Committee Decision No. 14; approval date: 25 March 2026). The requirement for written informed consent was waived because of the retrospective design and the use of de-identified archival data, as approved by the Ethics Committee.

### 2.7. Generative AI Assistance

During preparation and revision of the manuscript, the authors used ChatGPT (OpenAI, San Francisco, CA, USA) to assist with English-language editing, clarity, readability, and organization of selected sections. The tool was not used to generate primary study data or perform laboratory analyses. All AI-assisted output was reviewed and edited by the authors, who take full responsibility for the content of the manuscript.

## 3. Results

### 3.1. Patient Characteristics

The study cohort comprised 126 patients with cytologically evaluated thyroid nodules. The median age was 48.0 years (IQR, 38.3–57.0), 97 patients (77.0%) were female, and the median nodule diameter was 17.0 mm (IQR, 12.0–25.0). Surgical resection was performed in 69 patients (54.8%), of whom 32 (46.4%) had malignant final histopathology and 37 (53.6%) had benign/NIFTP diagnoses. Age, sex, and nodule size did not differ significantly between resected and non-resected patients. Surgical selection was strongly associated with TBSRTC category (*p* < 0.001; Table 1). Because molecular results were available before surgical decision-making, the histology-referenced performance cohort should be regarded as surgery-enriched and subject to verification bias.

### 3.2. Molecular Findings

BRAF, KRAS, and NRAS were tested in all 126 patients, whereas TERT promoter analysis was performed in six clinically selected cases, including four resected nodules. Overall, 35 of 126 patients (27.8%) harbored at least one BRAF/KRAS/NRAS mutation. BRAF was the most common alteration (18/126, 14.3%): 17 nodules harbored BRAF V600E and one harbored BRAF K601E. BRAF positivity showed the strongest gradient across TBSRTC categories, reaching 100% in TBSRTC category VI (*p* < 0.001; Table 2).

In the resected cohort, any BRAF/KRAS/NRAS mutation was detected in 23 of 32 malignant nodules (71.9%) and 10 of 37 benign/NIFTP nodules (27.0%; *p* < 0.001). BRAF alterations provided the clearest overall separation, occurring in 16 malignant nodules (50.0%) but only one benign/NIFTP nodule (2.7%; *p* < 0.001). Notably, the single BRAF-positive benign/NIFTP case was a TBSRTC category V nodule harboring BRAF K601E rather than V600E. KRAS and NRAS alterations were observed in both malignant and benign/NIFTP nodules and were not significantly associated with malignancy when analyzed individually (Table 3).

### 3.3. Malignancy Rates by TBSRTC Category and Mutation Status

Mutation positivity identified a high-risk subgroup within TBSRTC category III: 15 of 18 mutation-positive nodules (83.3%) were malignant, compared with 8 of 24 mutation-negative nodules (33.3%). In TBSRTC category IV, 3 of 8 mutation-positive nodules (37.5%) were malignant, whereas none of the 9 mutation-negative nodules were malignant. The complete stratified pattern is shown in Figure 1.

### 3.4. Diagnostic Model Performance

Three logistic regression models were evaluated: cytology alone, molecular testing alone, and the combined model incorporating cytology and molecular testing. In the entire resected cohort, the combined model achieved an apparent AUC of 0.81 (95% CI, 0.71–0.90), compared with 0.70 for cytology alone and 0.72 for molecular testing alone. At the Youden-optimal cutoff, the combined model had 91.9% specificity, 86.4% PPV, and 76.8% overall accuracy (Table 4). The paired bootstrap AUC improvement over cytology alone was 0.10 (95% CI, 0.03–0.18; *p* = 0.009). The optimism-corrected AUC was 0.78, and the mean repeated five-fold cross-validated AUC was 0.74.

Within the clinically most relevant indeterminate subgroup (TBSRTC category III–IV, *n* = 59), the combined model outperformed cytology alone in apparent discrimination. The apparent AUC increased from 0.65 to 0.81 (paired bootstrap ΔAUC = 0.15; 95% CI, 0.01–0.29; *p* = 0.036), while specificity increased from 42.4% to 90.9% and PPV increased from 54.8% to 83.3%. However, sensitivity of the combined model was 57.7%, underscoring its limited rule-out capability. The combined model had an optimism-corrected AUC of 0.79 and a mean repeated five-fold cross-validated AUC of 0.72 (Table 5). These estimates characterize the surgery-enriched, histologically verified subgroup and should not be interpreted as population-based performance estimates for unselected TBSRTC category III–IV nodules.

In the primary TBSRTC category III–IV combined model, mutation positivity remained independently associated with malignancy after adjustment for TBSRTC category (OR, 12.36; 95% CI, 2.93–52.05; *p* < 0.001). TBSRTC category IV showed lower observed odds of malignancy than category III (OR, 0.086; 95% CI, 0.015–0.476; *p* = 0.005), consistent with the non-monotonic category distribution and surgical selection pattern described below (Table 6).

Figure 2 provides a separate sensitivity analysis using continuous TBSRTC category coding and a single stratified 10-fold cross-validation partition. It is retained to illustrate the ROC curves and the effect of alternative TBSRTC parameterization; it is not the source of the primary internal-validation estimates. The primary categorical/dummy-coded models, evaluated with 500-resample bootstrap optimism correction and 100-repeat stratified five-fold cross-validation, are reported in Table 4 and Table 5.

### 3.5. Clinical Utility and Calibration

Exploratory decision curve analysis suggested that the combined model provided greater net benefit than cytology alone over portions of the examined 20–50% threshold range, particularly in the TBSRTC category III–IV subgroup (Figure 3). Because treatment thresholds were not prospectively defined and the analysis was restricted to operated nodules, these findings are hypothesis-generating and do not directly demonstrate fewer operations.

Apparent calibration plots showed general agreement between predicted and observed malignancy frequencies in both the entire resected cohort and the indeterminate subgroup (Figure 4); the corresponding Brier scores were 0.165 in both analyses. Hosmer–Lemeshow-type tests did not identify gross miscalibration (χ^2^ = 0.62, *p* = 0.43 and χ^2^ = 0.12, *p* = 0.73, respectively). Given the small sample and grouped nature of these assessments, calibration findings should be considered descriptive rather than evidence of transportability [16].

### 3.6. TBSRTC Parameterization and Category-Specific Performance

A notable feature of the resected cohort was a non-monotonic relationship between TBSRTC category and malignancy. TBSRTC category III nodules had a malignancy rate of 54.8% (23/42), whereas category IV nodules had a rate of 17.6% (3/17; Fisher’s exact *p* = 0.011). This pattern should not be interpreted as evidence that TBSRTC category IV is intrinsically less risky than category III. It most likely reflects selection and verification bias, the small category IV sample with only three malignant cases, the follicular composition of this subgroup, and potential interobserver variability. The observed category-specific malignancy proportions are therefore cohort-specific and should not be compared directly with population-based TBSRTC risk estimates.

Category-specific evaluation further demonstrated heterogeneity in the clinical value of the mutation panel. In TBSRTC category III, the panel achieved 84.2% specificity and 83.3% PPV, supporting a useful rule-in role. In TBSRTC category IV, the PPV was only 37.5%, consistent with the lower malignancy prevalence in the resected subgroup and the occurrence of RAS mutations in benign follicular-patterned lesions (Table 7). Because only three malignant category IV nodules were available, these category-specific estimates are unstable and should be interpreted cautiously.

## 4. Discussion

This single-center study provides preliminary evidence that targeted molecular testing adds information to thyroid FNA cytology, particularly in surgically verified TBSRTC category III–IV nodules. In the full resected cohort, the combined model improved apparent discrimination from an AUC of 0.70 with cytology alone to 0.81 (paired bootstrap ΔAUC = 0.10; 95% CI, 0.03–0.18). In the indeterminate subgroup, the increase from 0.65 to 0.81 was also supported by paired bootstrap analysis (ΔAUC = 0.15; 95% CI, 0.01–0.29) and was accompanied by higher specificity and positive predictive value. These estimates characterize a selected, surgery-enriched cohort in which molecular results were available before surgical decision-making and should not be generalized to all indeterminate thyroid nodules.

The principal clinical contribution of the combined model was not increased sensitivity, but improved rule-in performance. In the TBSRTC category III–IV subgroup, specificity increased to 90.9% and PPV to 83.3%; however, sensitivity was only 57.7%. This trade-off is clinically important: the restricted panel may help identify some nodules with a high probability of malignancy, but a negative or below-threshold result cannot reliably exclude malignancy. The assay should therefore be viewed as an adjunctive rule-in tool rather than a stand-alone rule-out test or an independent determinant of surgical management.

From a practical perspective, molecular testing should complement rather than replace established morphologic assessment. TBSRTC cytology remains the primary framework for initial cytopathologic risk stratification, while histopathology remains the definitive diagnostic reference standard in resected nodules. Molecular results should be integrated with cytomorphologic, ultrasonographic, and clinical findings, and a negative result from this restricted panel should not be used in isolation to defer further evaluation when other features remain concerning.

The magnitude and direction of these estimates are broadly compatible with the heterogeneous performance reported for molecular testing in indeterminate nodules, although direct cross-study comparison is limited by differences in cytological classification, assay breadth, disease prevalence, and surgical verification. Prospective multicenter validation studies of broader classifiers, including ThyroSeq v3 and the Afirma Genomic Sequencing Classifier, illustrate the substantially broader genomic or transcriptomic information available from contemporary platforms [19,20]. These studies are not directly comparable with the restricted Sanger panel used here because of differences in assay architecture, cohort assembly, and reference standards. In a recent single-center study of 105 indeterminate nodules, a 17-gene DNA/RNA NGS panel achieved 90% sensitivity, 87% specificity, 91% PPV, and 87% NPV among histologically evaluated cases [7]. In comparison, our combined model in the surgically verified TBSRTC category III–IV subgroup showed lower sensitivity (57.7%) but similar rule-in performance, with 90.9% specificity and 83.3% PPV. The lower sensitivity is biologically and technically plausible for a restricted hotspot panel that does not assess gene fusions or broader genomic alterations and reinforces that the present assay should be viewed as a rule-in adjunct rather than as a comprehensive rule-out classifier.

BRAF was the most informative individual alteration. Overall, BRAF positivity showed the strongest association with malignancy in the resected cohort. However, the biological interpretation of BRAF positivity was not uniform across all variants. One BRAF-positive TBSRTC category V nodule proved to be NIFTP on resection and harbored BRAF K601E rather than V600E, underscoring that non-V600 BRAF alterations may occur in follicular-patterned lesions and should not be interpreted identically to canonical BRAF V600E. Accordingly, the rule-in value of BRAF in this cohort appears to have been driven primarily by V600-type alterations.

RAS alterations showed a different and more context-dependent pattern. KRAS and NRAS hotspot mutations, including alterations affecting codons 12, 13, and 61, were identified in both malignant and benign/NIFTP nodules and were not individually associated with malignancy. This overlap is biologically consistent with the known occurrence of RAS-pathway alterations in follicular adenomas, NIFTP, and follicular-patterned carcinomas, and it explains the limited positive predictive value of the mutation panel in TBSRTC category IV nodules. A RAS-positive result should therefore be interpreted in conjunction with cytomorphology, imaging findings, and the overall clinicopathological context.

Assay architecture is therefore central to clinical interpretation. Broad DNA/RNA NGS classifiers can capture point mutations, fusions, copy-number changes, and other alterations at lower variant allele fractions, thereby offering greater potential for rule-out classification, but they require additional infrastructure, bioinformatic support, quality assurance, and financial resources [3,7,8]. The present Sanger-based approach uses routinely available FFPE cell-block material, permits H&E-guided enrichment by manual macrodissection, and avoids a separate prospectively collected FNA washout sample. These features may make it reproducible in laboratories with limited access to comprehensive NGS. Conversely, its restricted hotspot coverage, approximate 25% lesional-cell requirement, susceptibility to formalin-related DNA damage, and inability to detect fusions or copy-number alterations limit sensitivity. The recent Turkish washout-based BRAF/RAS study supports the feasibility of low-complexity testing [13]; our cell-block-based analysis is complementary because it evaluates archival material and quantifies incremental performance beyond TBSRTC cytology. Neither approach should be considered interchangeable with a validated broad genomic classifier.

*TERT* promoter analysis was performed in only six selected patients and in four resected nodules. Although two of three tested malignant nodules were *TERT*-positive, the sample was far too small and too selectively tested to support diagnostic-performance conclusions. The *TERT* findings should therefore be regarded as descriptive and hypothesis-generating rather than as a validated component of the primary molecular model.

The non-monotonic malignancy rates observed between TBSRTC categories III and IV require careful interpretation. The higher malignancy rate among resected category III nodules most likely reflects verification and referral bias. Molecular results were available before surgery and may have influenced the likelihood of resection; in addition, only a selected and clinically suspicious subset of category III nodules underwent surgery, whereas nearly all category IV nodules were resected. The category IV subgroup was also small, with only three malignant cases. This finding therefore characterizes the operated cohort and should not be interpreted as contradicting established TBSRTC risk estimates in broader thyroid practice.

Internal validation supported the direction of the main finding but also showed expected performance shrinkage. The combined model had optimism-corrected AUCs of 0.78 in the full resected cohort and 0.79 in the indeterminate subgroup; mean repeated five-fold cross-validated AUCs were 0.74 and 0.72, respectively. Exploratory decision curve analysis suggested possible net benefit within selected thresholds. These results require external validation before clinical implementation.

The present findings also highlight the importance of modeling TBSRTC categories appropriately. Treating TBSRTC category as a simple continuous scale may obscure non-linear and sample-specific relationships. Accordingly, TBSRTC category was modeled categorically in the primary analysis, while continuous coding was retained only as an illustrative sensitivity analysis in Figure 2.

Taken together, these findings support further evaluation of selective, context-aware targeted molecular testing in indeterminate thyroid nodules. Its potential value in this cohort was predominantly rule-in support, especially among surgically selected TBSRTC category III nodules, while non-V600 BRAF alterations and RAS positivity required cautious interpretation in follicular-patterned lesions. Compared with the recently reported large washout-based Turkish cohort [13], the present study addresses the complementary question of whether a low-complexity cell-block-based panel adds measurable information beyond cytology. The observed AUC gain and internal-validation results provide preliminary evidence of incremental diagnostic value, but not proof of improved patient outcomes, reduced surgery, or transportability to an unselected population.

### 4.1. Limitations

- The single-center, retrospective design and restriction of diagnostic-accuracy analyses to surgically resected nodules introduce substantial selection and verification bias. Importantly, molecular results were available before surgical decision-making and may have influenced which nodules proceeded to resection. Consequently, sensitivity, specificity, predictive values, and AUC estimates should be interpreted as cohort-specific and not as population-based performance estimates for unselected TBSRTC category III–IV nodules.
- The histopathologically verified sample was modest (*n* = 69). The TBSRTC category IV subgroup included only 17 resected nodules, of which three were malignant, limiting the stability of category-specific estimates; categories V and VI were also too small for reliable subgroup estimates.
- *TERT* promoter testing was performed in only six clinically selected cases and could not be incorporated meaningfully into the primary multivariable model.
- The targeted panel assessed BRAF exon 15 and KRAS/NRAS exons 2–4 but did not include gene fusions, copy-number changes, or broader genomic alterations. This restricted architecture limits sensitivity as a rule-out assay, and a negative result does not exclude malignancy. Because the panel was designed primarily for follicular cell-derived nodules, the findings should not be extrapolated to nodules with clinical or cytomorphological suspicion of medullary thyroid carcinoma, which require a dedicated diagnostic work-up.
- Bootstrap optimism correction and repeated cross-validation provide internal validation but do not replace independent external validation. In addition, the small number of outcomes per model parameter and sparse TBSRTC categories V–VI strata may make coefficients and performance estimates unstable [15,18].
- Long-term clinical outcomes and direct measurement of changes in surgical management were not available.

### 4.2. Future Directions

Prospective multicenter studies with standardized molecular testing, broader genomic panels, external validation, and integration of ultrasonographic and clinical variables are needed [11,12,18]. Future studies should also evaluate whether the improved specificity and net benefit observed here translate into fewer unnecessary operations without delaying treatment of clinically significant malignancy [10].

## 5. Conclusions

In this surgically verified, surgery-enriched single-center cohort, targeted BRAF/KRAS/NRAS testing provided incremental rule-in information beyond thyroid FNA cytology, with the largest observed increment in TBSRTC category III–IV nodules. BRAF V600E provided the strongest rule-in signal, whereas non-V600 BRAF variants and RAS hotspot mutations were not specific for malignancy and required interpretation within the broader morphologic and clinical context. The restricted panel had limited rule-out capability and should not independently guide surgical decision-making. Because of the selected cohort, modest sample size, and absence of external validation, these findings should not be generalized to unselected populations. Larger independent, preferably prospective multicenter cohorts are required before broader clinical implementation.

## Figures and Tables

**Figure 1:**
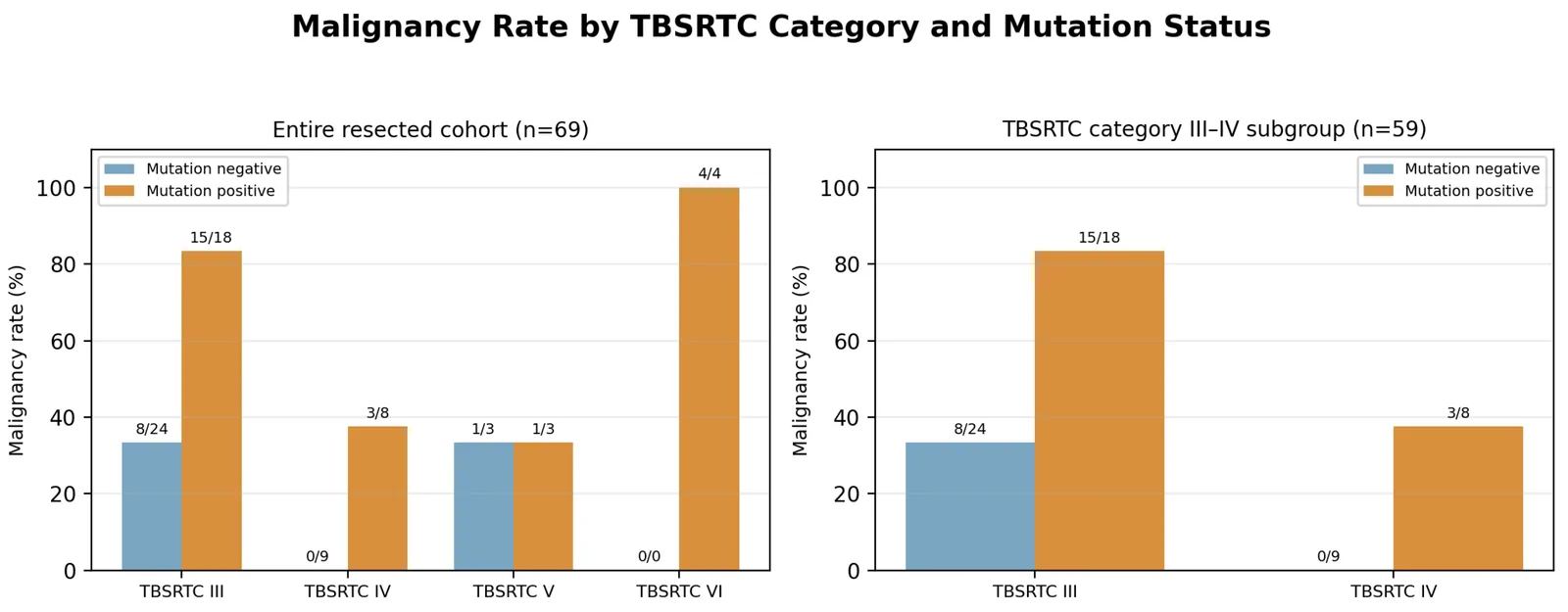
Malignancy rate by TBSRTC category and mutation status. Numbers above each bar indicate malignant/total nodules in the corresponding stratum. Left: entire resected cohort (*n* = 69). Right: indeterminate cytology subgroup (TBSRTC category III–IV, *n* = 59).

**Figure 2:**
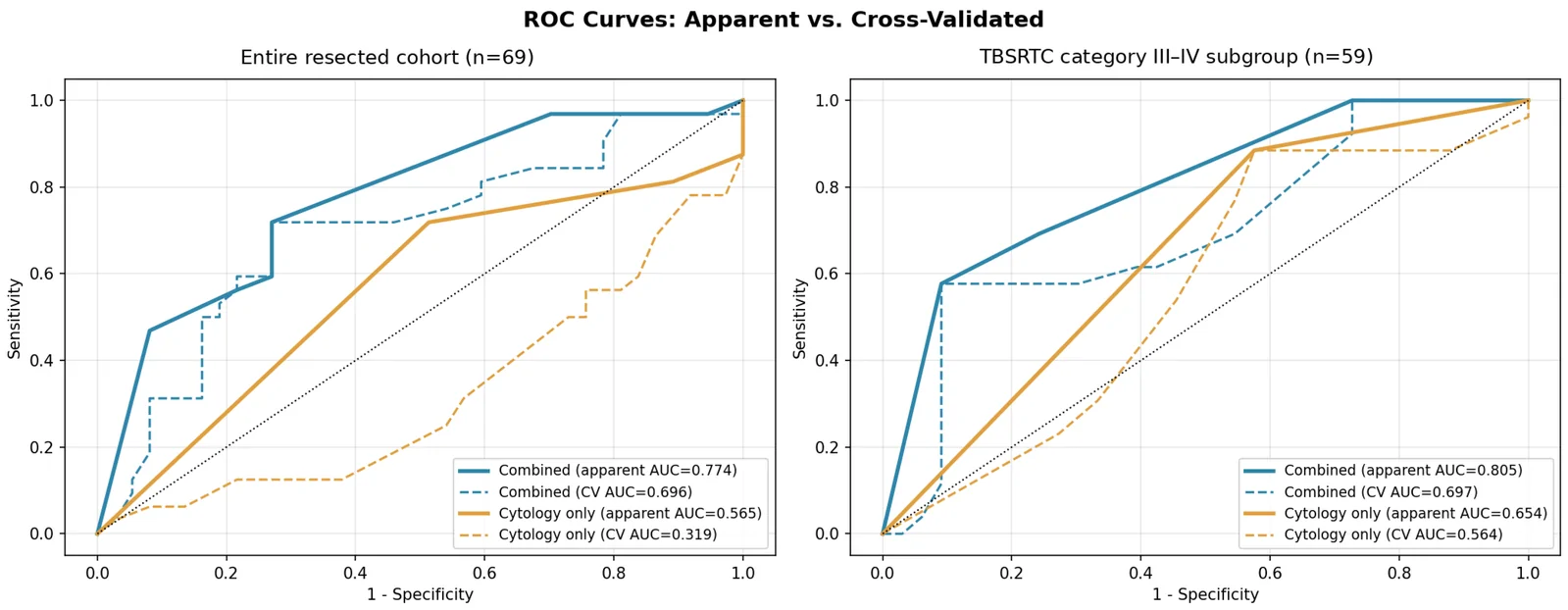
Apparent (solid) and single-partition stratified 10-fold cross-validated (dashed) ROC curves using continuous TBSRTC category coding as a sensitivity analysis. Left: entire resected cohort (*n* = 69). Right: indeterminate cytology subgroup (TBSRTC category III–IV, *n* = 59). These curves are illustrative and distinct from the primary dummy-coded models and repeated five-fold internal-validation estimates reported in Table 4 and Table 5.

**Figure 3:**
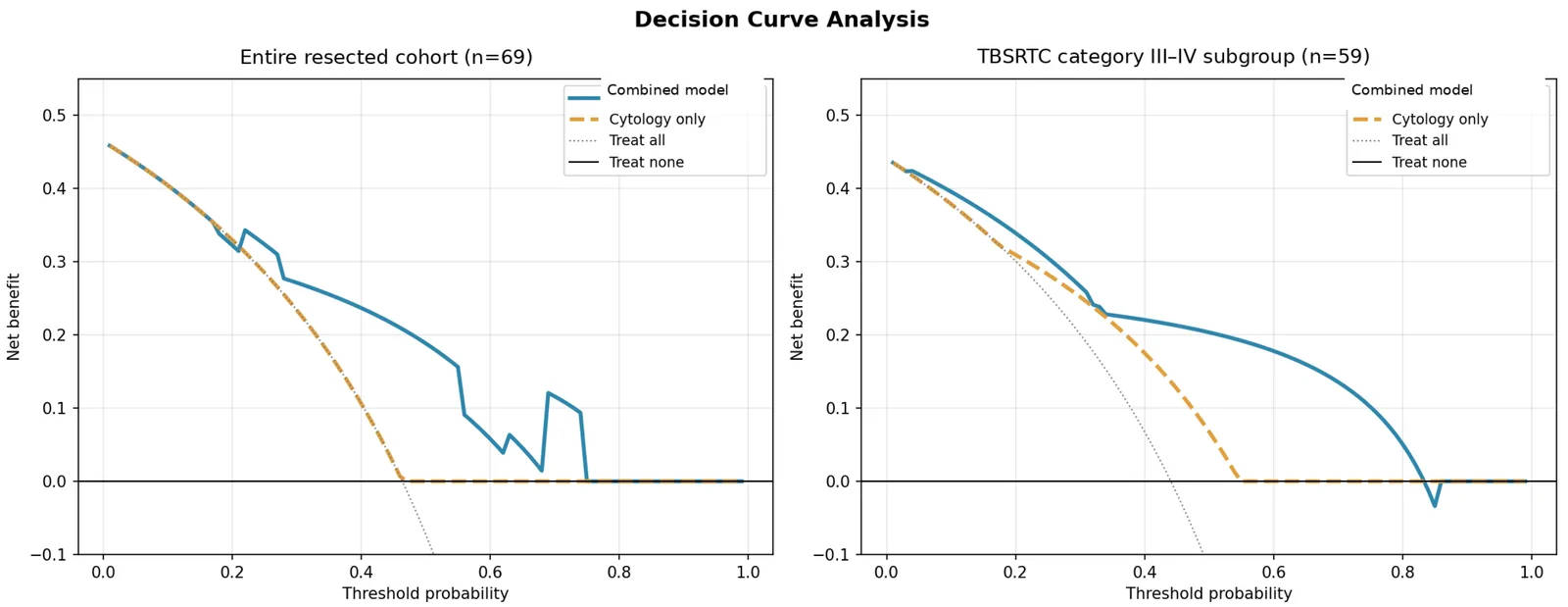
Decision curve analysis comparing the combined model incorporating cytology and molecular testing with cytology alone, treat-all, and treat-none strategies. Left: entire resected cohort. Right: TBSRTC category III–IV subgroup.

**Figure 4:**
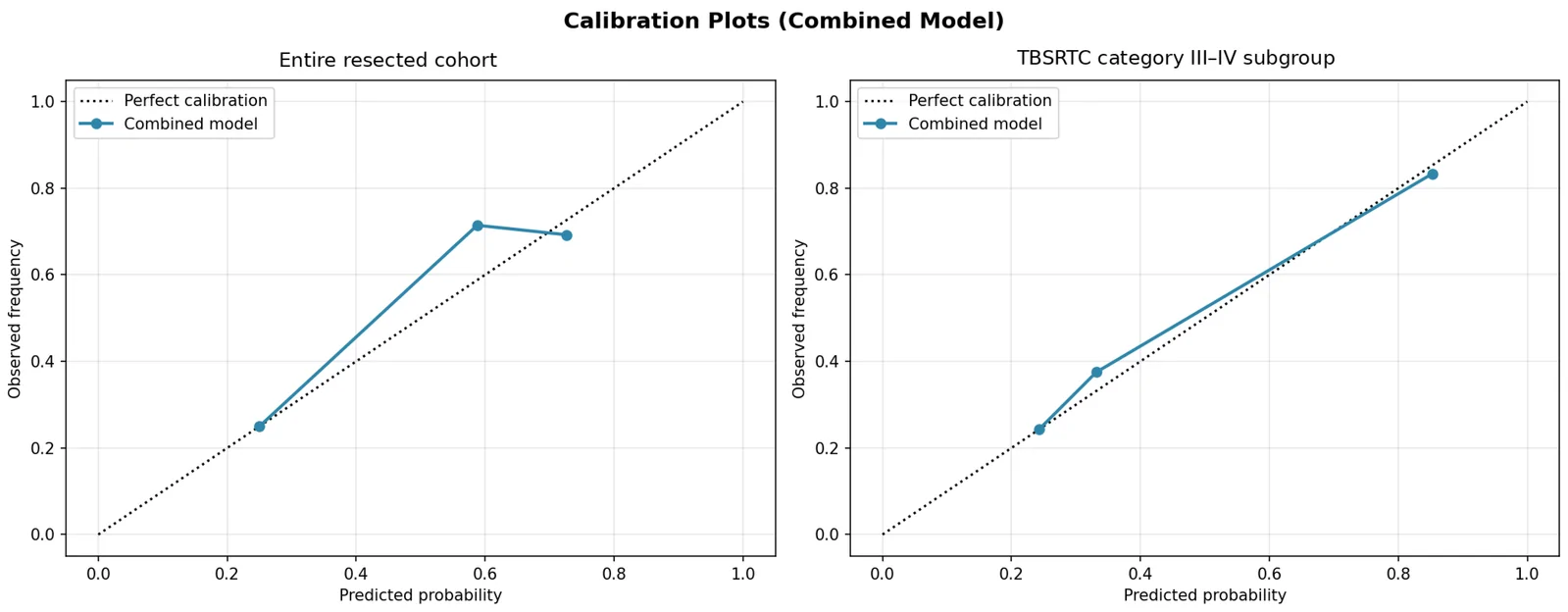
Calibration plots for the combined model incorporating cytology and molecular testing. Observed malignancy frequency is plotted against mean predicted probability within risk groups; the dotted line represents perfect calibration.

**Table 1:** Demographic and clinical characteristics of the study population.

Characteristic	Total (*n* = 126)	Resected (*n* = 69)	Non-Resected (*n* = 57)	*p*-Value
Age, years, median (IQR)	48.0 (38.3–57.0)	51.0 (43.0–58.0)	46.0 (35.0–56.0)	0.061
Female sex, n (%)	97 (77.0)	52 (75.4)	45 (78.9)	0.676
Nodule size, mm, median (IQR)	17.0 (12.0–25.0)	17.0 (12.0–25.0)	18.0 (12.0–24.0)	0.755
TBSRTC category III, n (%)	95 (75.4)	42 (44.2)	53 (55.8)	<0.001
TBSRTC category IV, n (%)	18 (14.3)	17 (94.4)	1 (5.6)	
TBSRTC category V, n (%)	8 (6.3)	6 (75.0)	2 (25.0)	
TBSRTC category VI, n (%)	5 (4.0)	4 (80.0)	1 (20.0)	
Final histopathology	—	37 benign/NIFTP; 32 malignant	—	—

Note. Age and nodule size were compared using the Mann–Whitney U test; sex was compared using Fisher’s exact test; and the TBSRTC distribution was compared using the Fisher–Freeman–Halton exact test. For TBSRTC category rows, percentages in the resected and non-resected columns are calculated using the total number of nodules within the corresponding category as the denominator; other percentages are calculated within the corresponding columns.

**Table 2:** Mutation frequency across TBSRTC categories in the entire cohort (*n* = 126).

Marker	TBSRTC III (*n* = 95)	TBSRTC IV (*n* = 18)	TBSRTC V (*n* = 8)	TBSRTC VI (*n* = 5)	Total (*n* = 126)	*p*-Value
*BRAF* (+)	10 (10.5)	2 (11.1)	1 (12.5)	5 (100)	18 (14.3)	<0.001
*KRAS* (+)	5 (5.3)	3 (16.7)	0 (0.0)	0 (0.0)	8 (6.3)	0.197
*NRAS* (+)	4 (4.2)	3 (16.7)	2 (25.0)	0 (0.0)	9 (7.1)	0.069
Any mutation	19 (20.0)	8 (44.4)	3 (37.5)	5 (100)	35 (27.8)	<0.001

Note. *p*-values were calculated using the Fisher–Freeman–Halton exact test. “Any mutation” denotes positivity for BRAF, KRAS, or NRAS. One BRAF-positive TBSRTC category V case harbored BRAF K601E; this nodule was resected and classified as NIFTP.

**Table 3:** Mutation frequencies in resected cases according to final histopathology (*n* = 69).

Marker	Malignant (*n* = 32)	Benign/NIFTP (*n* = 37)	*p*-Value
*BRAF* (+)	16 (50.0)	1 (2.7)	<0.001
*KRAS* (+)	2 (6.3)	6 (16.2)	0.270
*NRAS* (+)	5 (15.6)	3 (8.1)	0.457
*TERT* (+) *	2/3 (66.7)	0/1 (0.0)	1.000
Any mutation (+)	23 (71.9)	10 (27.0)	<0.001

Note. *p*-values were calculated using Pearson’s chi-square test or Fisher’s exact test, as appropriate. * TERT promoter analysis was performed in only four of the 69 resected patients (three malignant and one benign/NIFTP); percentages are relative to tested cases only. “Any mutation” denotes positivity for BRAF, KRAS, or NRAS. The single BRAF-positive benign/NIFTP case harbored BRAF K601E and was classified as NIFTP on final histopathology.

**Table 4:** Diagnostic performance of the three models in the entire resected cohort (*n* = 69).

Model	Apparent AUC (95% CI)	Repeated 5-Fold CV AUC	Sens	Spec	PPV	NPV	Acc
Cytology only	0.70 (0.59–0.80)	0.64	84.4%	48.6%	58.7%	78.3%	65.2%
Molecular only	0.72 (0.60–0.85)	0.66	71.9%	73.0%	69.7%	75.0%	72.5%
Cytology and molecular testing	0.81 (0.71–0.90)	0.74	59.4%	91.9%	86.4%	72.3%	76.8%

Note. AUC = area under the ROC curve; CV = repeated stratified five-fold cross-validation (100 repeats); Sens = sensitivity; Spec = specificity; PPV = positive predictive value; NPV = negative predictive value; Acc = accuracy. Performance metrics were calculated at the in-sample Youden-optimal cutoff and are descriptive. The combined model had an optimism-corrected AUC of 0.78 and a Brier score of 0.165.

**Table 5:** Diagnostic performance of the three models in the indeterminate cytology subgroup (TBSRTC category III–IV, *n* = 59).

Model	Apparent AUC (95% CI)	Repeated 5-Fold CV AUC	Sens	Spec	PPV	NPV	Acc
Cytology only	0.65 (0.55–0.76)	0.59	88.5%	42.4%	54.8%	82.4%	62.7%
Molecular only	0.72 (0.61–0.84)	0.66	69.2%	75.8%	69.2%	75.8%	72.9%
Cytology and molecular testing	0.81 (0.70–0.90)	0.72	57.7%	90.9%	83.3%	73.2%	76.3%

Note. CV denotes repeated stratified five-fold cross-validation (100 repeats). Performance metrics were calculated at the in-sample Youden-optimal cutoff for each model and are descriptive. The combined model had an optimism-corrected AUC of 0.79 and a Brier score of 0.165.

**Table 6:** Multivariable logistic regression for malignancy in the primary TBSRTC category III–IV cohort (*n* = 59).

Predictor	β	Odds Ratio	95% CI	*p*-Value
Intercept	−0.759	0.47	0.20–1.08	0.076
TBSRTC IV vs. III	−2.456	0.086	0.015–0.476	0.005
Any BRAF/KRAS/NRAS mutation	2.514	12.36	2.93–52.05	<0.001

Note. The outcome was malignant versus benign/NIFTP histopathology. TBSRTC category III and mutation-negative status were the reference categories. CI = confidence interval. The inverse category IV association reflects the selected operated cohort and should not be interpreted as a population-level TBSRTC risk estimate.

**Table 7:** Performance of the mutation panel within each TBSRTC category.

TBSRTC Category (n, Malignant)	Sensitivity	Specificity	PPV	NPV
III (*n* = 42; 23 malignant)	65.2%	84.2%	83.3%	66.7%
IV (*n* = 17; 3 malignant)	100%	64.3%	37.5%	100%
V (*n* = 6; 2 malignant)	50.0%	50.0%	33.3%	66.7%
VI (*n* = 4; 4 malignant)	100%	—	100%	—

Note. The numbers in TBSRTC categories V and VI were too small for stable estimates and are reported only for completeness. Category-specific estimates should be interpreted cautiously because of small subgroup sizes; in addition, one BRAF K601E-positive TBSRTC category V nodule was classified as NIFTP on final histopathology.

## Data Availability

The data presented in this study are available on reasonable request from the corresponding author. The data are not publicly available because of patient privacy and ethical restrictions.
